# Structural insights into functional amyloid inhibition in Gram −ve bacteria

**DOI:** 10.1042/BST20160245

**Published:** 2016-12-02

**Authors:** William Hawthorne, Sarah Rouse, Lee Sewell, Stephen J. Matthews

**Affiliations:** Department of Life Sciences, Imperial College London, South Kensington, London SW7 2AZ, U.K.

**Keywords:** amyloid inhibitors, curli, Fap, functional amyloid

## Abstract

Amyloids are proteinaceous aggregates known for their role in debilitating degenerative diseases involving protein dysfunction. Many forms of functional amyloid are also produced in nature and often these systems require careful control of their assembly to avoid the potentially toxic effects. The best-characterised functional amyloid system is the bacterial curli system. Three natural inhibitors of bacterial curli amyloid have been identified and recently characterised structurally. Here, we compare common structural features of CsgC, CsgE and CsgH and discuss the potential implications for general inhibition of amyloid.

## Amyloid

Amyloids are best known for their role in debilitating, degenerative diseases in humans, such as Alzheimer's disease and Parkinson's disease. In these cases, misfolded polypeptides aggregate to form amyloid fibres, these ordered structures form a cross-β-structure and are highly stable with resistance to degradation [1,2]. A broad range of proteins, both native and intrinsically disordered, have been shown to be capable of forming amyloid, and it has been suggested that amyloid represents a basic low-energy protein structure available to many different polypeptides [3]. Not all amyloid is the product of protein dysfunction, many species, including eukaryotes and prokaryotes, deliberately produce amyloid for functional purposes. The biological roles of these functional amyloids are diverse including protein storage, silk formation, melanin production, immune response and biofilm formation [4–8]. To enable the organisms to employ amyloid effectively, it is critical that they have mechanisms to control the process of amyloid formation and some functional amyloid systems even have their own dedicated secretion system (i.e. bacterial type VIII) [9]. The study of functional amyloid therefore has the potential to provide insights into the control of amyloid formation, which could assist in the treatment of amyloidosis diseases, the development of novel biomaterials and methods of biofilm dispersal in industry and medicine.

## Bacterial functional amyloid

**Figure 1.**
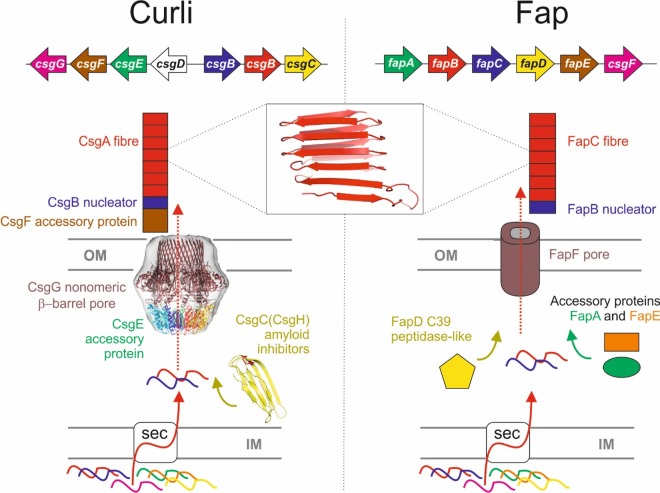
Schematic representation of the Curli and Fap amyloid systems. The two distinct biogenesis machineries from Gram −ve bacteria for the transport, secretion and assembly of the functional amyloids: Curli and Fap operons. Both systems are translocated via the Sec machinery into the periplasmic space between the inner membrane (IM) and outer membrane (OM). In some Curli operons, CsgC is not present and is replaced with the distantly related CsgH gene, which performs an identical function by inhibiting curli amyloid formation in the periplasm. The Fap operon is less well characterised, but FapA and FapE appear to be accessory proteins that may also control amyloid formation. The predicted structure of the amyloid fold for CsgA subunits is shown and evidence suggests that FapC subunits adopt a similar architecture.

The curli amyloid of *Escherichia coli* is the best-characterised functional amyloid system [10]. Curli fibres are attached to the cellular surface and contribute to surface adhesion and biofilm formation [11]. The curli system is encoded by two divergently transcribed operons *csg*BAC and *csg*DEFG (Figure 1) [12]. The *csg*BAC operon produces both of the fibre components CsgB and CsgA as well as CsgC, which has been recently discovered to be a potent inhibitor of amyloid fibrillation [13]. The CsgA protein contains five repeats (S–X5–Q–X–G–X–G–N–X–A–X3–Q) that have been shown to be amyloidogenic, CsgB; the nucleator has four similar repeats (A–X3–Q-X–G-X2–N–X–A–X3–Q) followed by a less well-conserved fifth repeat with four positively charged residues that anchor CsgB to the cell surface [14–16]. In the amyloid, these are believed to form a β-turn-β secondary structure that is arranged into the cross-β-structure in which glutamine and asparagine residues align and stabilise the fold [14,17,18]. The effects of the individual repeats have been studied in detail and they appear to play different roles in amyloid assembly. Repeats 1, 3 and 5 are amyloidogenic in isolation, whereas repeats 2 and 4 are not [19]. Repeats 1 and 5 are required for curli fibrillation *in vivo* and cannot be seeded by CsgA or CsgB *in vitro* [20]. The differences between the repeats have been further dissected using simulations to study the dimerisation properties of peptides corresponding to the repeats; these showed that hydrogen bonding appeared to be the main stabilising force between the repeats and that the position of the β-hairpin in R4 has a major impact on its ability to dimerise correctly [21]. It was also shown that the sequence of CsgA contains what have been described as ‘gatekeeper’ residues, which inhibit spontaneous amyloid formation and provide a form of intramolecular control [20].

The *csg*DEFG operon encodes the biosynthetic machinery required for the export and assembly of the fibre as well as the regulator for the *csg*BAC operon CsgD. The curli fibres themselves are composed primarily of CsgA, which is secreted to the extracellular space where fibre formation is nucleated by CsgB in a mechanism that has been described as nucleation–precipitation, since unfolded subunits in solution are nucleated by folded subunits causing them to assemble and form fibres, and as Type VIII secretion, differentiated from other types of secretion by the fact that fibre extension occurs extracellularly [9,22]. The secretion of these amyloid components involves the CsgEFG complex located in the bacterial outer membrane, and the structure of the transmembrane channel adopts a novel β-barrel assembled from a nonameric oligomer of the CsgG protein [23]. A combination of NMR and EM has also provided evidence for the arrangement of CsgE within the complex, in which it forms a 9:9 complex and caps the periplasmic opening of the channel [23,24]. CsgE interacts with the CsgA amyloid and is likely to play a role in recognising the substrates for transport. CsgF is secreted and associates with the extracellular side of CsgG; there it is required for the anchoring of CsgB to the membrane and is believed to be positioned on the extracellular side of the barrel [25,26].

Interestingly, a genetically unrelated but morphologically similar system of functional amyloid has been identified and described as functional amyloid in *Pseudomonas* (Fap) [27]. The Fap system is encoded by a single operon *fap*ABCDEF with several components being functionally analogous to those of the Curli system and presumably forming another Type VIII secretion system (Figure 1) [27]. FapC is the primary component of the fibre with FapB implicated as the nucleator. Similar to CsgA and CsgB, these also contain sequence repeats (X15–G–X4–N–X3–G–X6–N–X7); however, they have three rather than five copies [27,28]. Although the display difference, both curli and *Pseudomonas* amyloid repeats share a Q/N–X10–Q/N motif, which is perhaps important in defining the similarities in the resulting fibres. It is unknown whether FapC contains gatekeeper residues analogous to those contained in CsgA. Unlike the curli system, FapC is known to contain a C–X–X–C motif at its C-terminus that may be involved in redox reactions, perhaps between FapC molecules or with the FapE putative accessory protein, which also contains a cysteine residue. FapF is predicted to be a β-barrel that is likely to provide the channel through which the amyloid is secreted. FapA, FapD and FapE appear to be accessory proteins with unknown functions [27].

## Natural amyloid inhibitors of the curli system

The curli system possesses two proteins capable of inhibiting fibrillation of the CsgA amyloid *in vitro*, namely CsgC and CsgE. CsgC is capable of inhibiting CsgA fibrillation at substoichiometic ratios and even as low as 1:1000, whereas CsgE inhibits at a 1:1 ratio [13,25]. Additionally, CsgC has been shown to possess a functional homologue in many bacteria, known as CsgH, which is genetically distinct to CsgC, but adopts a very similar three-dimensional structure and inhibits curli amyloidogenesis equally potently [29]. Both CsgH and CsgC appear to transiently interact with the CsgA monomers via electrostatically driven encounters [29]. It has been suggested that CsgC delays amyloid fibrillation by perturbing a subset of the CsgA-disordered ensemble that is competent for forming amyloid and thereby redirecting it back into either a more unfolded state or an off-pathway oligomer [13,29]. This mode of amyloid inhibition is akin to the plate spinner in the circus art of ‘plate spinning’. CsgC/H interacts transiently with a low population critical conformation (i.e. ‘*brief tending of individual wobbling plates*’) and diverts this back into an expanded conformational pool (i.e. ‘*gets the plate spinning again*’) and away from amyloid assembly. CsgE is likely to perform a slightly different role and interacts with CsgA molecules more specifically and tightly, which keeps it in a state that facilitates the final handover to CsgG for secretion. CsgE may simply act as a traditional chaperone, simply binding individual CsgA molecules and capturing a high-energy state thereby preventing stable amyloid formation and priming them for secretion. CsgC and CsgH have been shown to inhibit the assembly of other amyloids *in vitro*, including the human α-synuclein and the *Pseudomonas* functional amyloid [13,29]. Interestingly, CsgE does not inhibit α-synuclein *in vitro*, but actually appears to accelerate fibrillation [30]. It has been suggested that this may be the result of the CsgE oligomer promoting intermolecular interactions [25]. The small molecule amyloid inhibitor 2-pyridone FN075 is also capable of inhibiting CsgA aggregation, but stimulates α-synuclein fibrillation [31]. Since FN075 has been shown to direct the formation of oligomers of CsgA and α-synuclein, it is tempting to speculate that oligomeric CsgE also inhibits CsgA in this way, with a nonameric CsgE oligomer binding a stoichiometric quantity of CsgA and inducing oligomerisation that does not proceed to amyloid. Here, we compare the structure features of the natural curli amyloid inhibitors CsgC, CsgH and CsgE.

## Comparison of CsgC and CsgH

**Figure 2.**
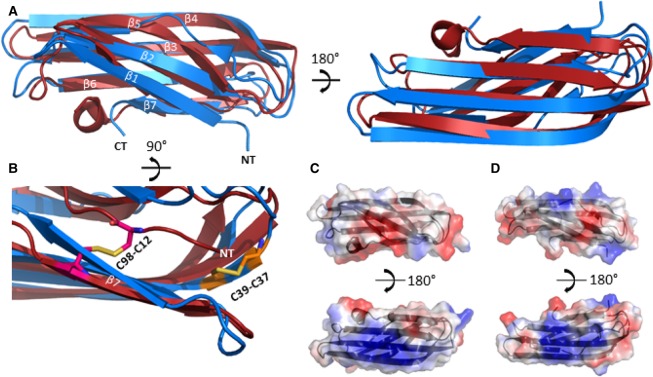
Comparison of the three-dimensional structures of CsgC and CsgH. (**A**) Cartoon representation of the superposition of CsgH (red) (PDB code 2N59) and CsgC (blue) (PDB code 2Y2T) with the aligned β-strands and the termini labelled, illustrating the similarity between the two structures. (**B**) Cartoon representation of the superposition of CsgH and CsgC highlighting the differing positions of the disulphide bridges shown as sticks. (**C**) Diagram illustrating the electrostatic surface potential of CsgH with the positive patches shown in blue and the negative in red. (**D**) Diagram illustration of the electrostatic surface of CsgC with the positive patches shown in blue and the negative in red.

We previously elucidated the atomic resolution structures of CsgC and CsgH using X-ray crystallography and NMR, respectively [29,32]. CsgC and CsgH share a low sequence identity (∼16%), but have similar tertiary structures with an RMSD between the protein backbones of ∼2.5 Å. The proteins form seven-stranded immunoglobulin-like β-sandwich structures (Figure 2A). It is possible that β-sheets are important for interaction with transient β-structure, formed within amyloid-prone monomers in the fibrillation pathway. Both proteins also contain disulphide bridges. CsgC possesses a CxC motif, while CsgH has the cysteines more distantly spaced which serve to pin the C-terminal and N-terminal β-strands together (Figure 2B). The role of the cysteines is unclear and their position in the structures is not conserved between the two proteins. The cysteines are likely to have a structural role in CsgH, but presumably do not serve a similar function in CsgC. It has been observed that CsgC possesses reduction potential similar to a disulphide isomerase; however, the biological importance of this is unknown [32].

Electrostatic properties have been suggested as playing an important role in the mechanism of several amyloid inhibitors, including CsgC and CsgH [25,29,33]. Significantly, the surface electrostatics of CsgC and CsgH are well conserved; in particular, there is a strong positively charged patch on the surface of the protein corresponding to the β3 and β4 strands, and there are also negatively charged patches neighbouring this (Figure 2C). Mutations of charged residues have been shown to affect the ability of both proteins to inhibit amyloid fibrillation [29].

In eukaryotes, there are other small, immunoglobulin-like chaperone proteins capable of inhibiting diverse aggregating proteins at sub-stochiometiric ratios via transient interactions; they are also thought to redirect misfolded protein back into a more unfolded state or off-pathway oligomer. The Ig-like structure of these small heat shock molecular chaperone proteins (sHsps [34]) is important for function, and the mobile C-terminus of the sHsps has been shown to be important for stabilising both the protein and the complexes it forms. There are differences between these bacterial amyloid inhibitors as sHsps inhibit via important hydrophobic interactions, which were not implicated in mutagenesis studies of the CsgC/CsgH mode of action [29].

## Solution structure of CsgE

**Figure 3.**
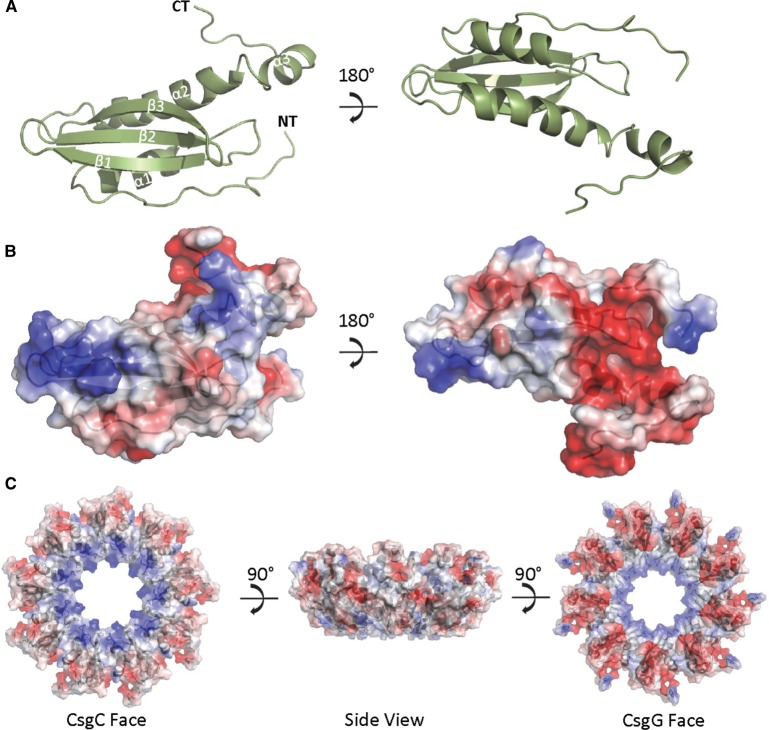
Structure of CsgE. (**A**) Cartoon representation of the crystal structure of CsgE (green) (PDB code 2NA4) with the secondary structure elements and the termini labelled. (**B**) Diagram illustrating the electrostatic surface potential of CsgE with the positive patches shown in blue and the negative in red. (**C**) Diagram illustrating the surface potential of the putative CsgE nonamer as modelled using Symmdock [33] with the positive patches shown in blue and the negative in red; the model is shown in subpanels in three orientations illustrating the opposing electrostatics of the ‘CsgC view’ and the ‘CsgG view’, as well as the view of the complex from the side.

The NMR structure of a non-oligomerising mutant of CsgE (W48A/F79A) has been recently determined [24]. Unlike CsgH and CsgC, CsgE possesses an anticodon-binding domain-like fold rather than the IG-like fold, and it does not possess any cysteine residues (Figure 3A). Although the full structure of CsgE does not align well with CsgC, it is notable that CsgE shares contain an exposed β-sheet face that is reminiscent of those in CsgC and CsgH. Notably, CsgE also possesses a striking electrostatic distribution, with similar patches of conserved positive charge on one side of molecule. This distribution can be described as being a positive, periplasmic- or CsgC-facing bottom with a negative, CsgG-facing top. It has been suggested that the negative surface interacts with CsgG, while the concentrated positive charge is involved in interacting with the substrate (Figure 3B) [25]. Taken together, the three structures indicate that positive charge is crucial for the interaction with CsgA and its safe guidance to the secretion system. The positively charged region is highly conserved in CsgC and CsgH, and electrostatic mutations have a severe impact on their ability to inhibit amyloid. A model of the CsgE nonamer was calculated from the deposited monomer CsgE structure [24] using Symmdock [35] using restraints that maintain monomerising mutations near the interface. The highest scoring complex based of geometric complementarity (Figure 3C) is consistent with the notion that CsgE oligomer presents a contiguous, positively charged surface for recruitment of the negatively charged CsgA. The differences between CsgE and CsgC, such as the more expansive positively charged surface on the β-sheet of CsgC and CsgH and their monomeric states, are likely to underlie the differences in amyloid inhibition efficiency between CsgE and CsgC.

CsgC and CsgH have been shown to be capable of effectively inhibiting other amyloids, including both the human amyloid α-synuclein and the *Pseudomonas* functional amyloid FapC [29]. The cross-species reactivity of these inhibitors suggests that the mechanism is not sequence specific. Whether CsgE is also capable of inhibiting FapC is unknown, but when tested, it was found to stimulate rather than inhibit α-synuclein fibrillation [30], probably through an increase in local concentration that results from the nonameric nature of native CsgE. The functions of the other *fap* operon genes remain unclear; however, from our knowledge of the curli operon and given the innate toxicity of unmanaged amyloid, it seems likely that one or more of the proteins must be capable of controlling amyloid formation. Out of the six genes in the operon, three of the proteins are known to be secreted and be part of or associated with the external fibre; these include FapC, FapB and FapE. This leaves the three other gene products as the main candidates for amyloid inhibition. FapA is a protein of unknown function, but it was shown that a knockout led to alterations in fibre composition where the fibres become predominantly composed of FapB rather than FapC [27,28]. This could be interpreted as evidence that FapA inhibits fibrillation of FapC and in its absence, the protein is no longer secretion competent, although it could equally reflect another regulatory role. FapD is a structured protein from the C39-like peptidase family; commonly found in ABC-transporter systems, these are often involved in bacteriocin processing and Quorum sensing and are known to cleave a double-glycine motif. FapD is predicted to have the catalytic residues necessary for the protease activity and the secretion pore FapF contains a double-glycine motif, but this activity has yet to be ascertained [27,28,36]. It has been observed that some C39-like peptidases are inactive proteolytically and instead serve a role in substrate handing and recognition. It is conceivable that this function is exploited in a chaperone/inhibitory function for FapD [37]. FapF is a predicted β-barrel protein that is likely to provide the channel through which the amyloid components are secreted. Structure predictions suggest that a long N-terminal extension exists within the periplasm, and it is interesting to note that this region contains a significant number of charged residues, which could provide an important platform for electrostatic interactions that may help to recruit Fap substrates as well as inhibit amyloid formation and drive secretion, akin to CsgE in the curli system. A further study of the *Pseudomonas* operon and elucidation of the molecular mechanisms underlying this alternative Type VIII secretion system will be invaluable for our understanding of the principles underlying the control of amyloid in nature.

## Abbreviations

Fap: functional amyloid
IG: immunoglobulin
RMSD: root-mean-squared deviation
sHsps: small heat shock molecular chaperone proteins
